# Clinical and Socio-demographic Profile of the First 33 COVID-19 Cases Treated at Dedicated Treatment Center in Ethiopia

**DOI:** 10.4314/ejhs.v30i5.2

**Published:** 2020-09

**Authors:** Sisay Teklu, Menbeu Sultan, Aklilu Azazh, Aschalew Worku, Berhane Redae, Miraf Walelegn, Muluwork Tefera, Rahel Argaw, Woldesenbet Waganew, Sisay Yifru, Wondwossen Amogne, Natinael Tssema, Abebaw Bekele, Yonas Gebregziabher, Hiruy Araya, Addisu Birhanu, Getachew Demoz, Bethlehem Tadesse, Yakob Seman, Aschalew Abayneh

**Affiliations:** 1 Addis Ababa University, College of Health Sciences; 2 Saint Paul's Hospital Millennium Medical College; 3 Jhpiego Ethiopia; 4 Federal Ministry of Health; 5 Save the Children Ethiopia; 6 Eka-Kotebe Hospital; 7 Ethiopian Public Health Institute

**Keywords:** COVID-19, SARS CoV2, Pandemic, Ekka-Kotebe, Ethiopia

## Abstract

**Background:**

Severe respiratory tract infection caused by family of Corona viruses has become world pandemic. The purpose of this study was to describe the first few COVID 19 cases in Ethiopia.

**Method:**

Descriptive study was conducted on the first 33 consecutive RT-PCR confirmed COVID 19 cases diagnosed and managed at Ekka-Kotebe COVID Treatment Center in Addis Ababa, Ethiopia.

**Result:**

The median age of the cases was 36 years. Cough, headache and fever were the most frequent symptoms. Diarrhea, sore throats, loss of taste and/or smell sensation were among the rare symptoms. Most (84.8%) had mild to moderate disease, and 15.2%(n=5) were critical at the time of admission. Among the five ICU admissions, four patients required invasive mechanical ventilation. Thirty cases were discharged after two pairs of nasopharyngeal and oropharyngeal samples turned negative for SARS CoV2. Three cases from the ICU died while on mechanical ventilator. The age of the two deaths was 65 years, and one was 60 years. With the exception of three, all cases were either imported from abroad or had contact with confirmed cases.

**Conclusion:**

Most of our patients were in the younger age group with male predominance and few with comorbidities. Cough was the commonest symptom followed by headache and fever. As it was in the early stage of the pandemic, observation of more cases in the future will reveal further clinical and demographic profiles of COVID-19 cases in Ethiopia.

## Introduction

Pneumonia of unknown cause detected in Wuhan China was first reported to the WHO country office on December 31/2019. The outbreak was declared a Public Health Emergency of International Concern on January 30/2020. The World Health Organization (WHO) used the term COVID-19 to refer to acute respiratory infection caused by family of corona virus initially seen in Wuhan, China (1). Currently, the virus that causes COVID 19 is referred to as Severe Acute Respiratory Syndrome corona virus 2 (SARS CoV2), by the International Committee on Taxonomy of Viruses (2).

The report of WHO-China Joint Mission, deployed to assess the outbreak of respiratory infection of unknown etiology found that COVID-19 is a zoonotic disease. Viral Bioinformatics analyses indicated that the virus has features typical of the coronavirus family belonging to the Beta coronavirus 2B lineage. In this report, the median age of the affected people was 51 years, and the report revealed that the virus is transmitted via droplets and fomites (3). On March 11/2020, the WHO Director General declared that COVID-19 is global pandemic (4).

The recent COVID-19 pandemic is one of the rapidly spreading infectious disease epidemics the world has witnessed. Initially seen as a single case in Hubie Province, Wuhan City in China, it has reached all countries in the world with in three months (5). Like the infectious epidemics in the past, it followed the tourist and business routes to cross borders and reach almost all countries. Up to April 26/2020, nearly 3 million confirmed cases and 206,000 deaths were reported throughout the world (6). The spread of the disease and the damage it is causing vary from country to country. Early introduction of preventive public health measures proved to reduce the burden of the disease and fatality.

There are limited reports on this new disease. The available reports are mostly from China where the outbreak was initially reported and most evidences are from case serieses. Like other respiratory tract infections, hand hygiene and protecting the nose and face by face mask are believed to protect transmission. However, recent reports revealed that the virus is also detectable in the GI secretions. The incubation period varies from 2 to 14 days. The virus has been isolated from respiratory secretions prior to development of symptoms in pre-symptomatic patients, making prevention of transmission very difficult. Clinical manifestations vary from asymptomatic (pre-symptomatic) to respiratory failure (7).

A report on 1099 confirmed COVID-19 cases from 552 hospitals in 30 different regions of mainland China showed that the most common symptoms were fever (43.8% on admission, and 88.7% during hospitalization) and cough (67.8%) and diarrhea (3.8%). In this report, most cases were mild disease. Severe diseases was reported in elderly and in those with comorbidity. Fifteen patients died of the disease (8).

Another study from India showed that most laboratory confirmed cases were male, symptomatic, had travel history out of India. The majority were young, twenty-nine percent of patients had comorbidities and most recovered from the disease. The common symptoms observed were fever and cough (9). Next to China, Italy became the epicenter for COVID in Europe (10).

A retrospective study done in 1591 Intensive Care Unit (ICU) admitted cases in Lombardy, Italy, showed that the majority of cases were old with median age of 63 years. The majority of patients needed respiratory support whereas 88% (n=1150) needed invasive mechanical ventilation while 26% (n=405) of cases died (11).

The Ethiopian Federal Government started to mobilize all governmental bodies and started readiness for the epidemics since the first case in China was reported. The Federal Ministry of Health and the Ethiopian Public Health Institute (EPHI) were given full authority in preventing, testing, reporting and treating COVID-19 in Ethiopia. The initial measures taken were screening all arrivals from COVID-19 affected countries at Bole International Airport for the presence of fever, collecting their address and checking for the development of any COVID symptoms for two weeks by telephone. The first case was diagnosed on March 13, three days after he arrived from Burkina Faso. All his contacts were traced and quarantined. Contact tracing and quarantine and isolation of patients with COVID-19 symptoms visiting health facilities were started at the same time.

National preparedness plan introduced in the country includes alerting all the regions about the epidemics, educating the people on social distancing, hand hygiene and avoiding mass gathering at market places, social events and religious places. The mass media focused on health education and expert advice on most television and audio channels was introduced. Frequent free text messages about COVID and COVID-19, alerting voice messages during all telephone calls, was put in place by telephone service provider throughout the country. Hand washing at public places like bus and train stations and at the gate of banks and supermarkets was introduced starting from early March. The purpose of this small scale dscriptive study was to give initial information to scientific community in the region about the cases of COVID 19 in Ethiopia.

## Materials and Methods

This study is an observational descriptive study on 33 COVID-19 RT-PCR confirmed cases admitted to and managed at Ekka-Kotebe COVID-19 Treatment Center. The cases in this study include all cases that had an outcome until April 20/2020, including the first case ever diagnosed in Ethiopia on March 13, more than two months after the first case reported in Wuhan China. The study describes the sociodemography, clinical presentation and treatment outcome of the cases.

All suspected cases and those with contact with confirmed cases were tested using RT PCR initially at the EPHI laboratory, but subsequently, more laboratories were opened and the number of tests done every day increased. Sample collection, transport and testing followed standard COVID-19 sample collection, transport and testing techniques recommended by WHO. Treatment of confirmed cases was using the recommendations by the The National Comprehensive COVID-19 Management Guideline.

RT-PCR confirmed COVID-19 cases are triaged at the triage area at the Ekka-Kotebe Treatment Center according to the national triage criteria. Mild and moderate cases are admitted to the General Ward, severe cases to Severe Cases Ward and critical cases to the ICU. All cases were treated with chloroquine sulphate 500mg PO BID for five days and Azithromycin 500mg PO/day for three days. Oxygen therapy was provided based on the oxygen saturation and respiratory condition of the patient. Intravenous Ceftriaxone was given for all moderate, severe and critical cases. Fever and headache were managed with paracetamol (acetaminophen) 1gm PO every 6 hours as required. The discharge criteria used in the center is complete resolution of symptoms and negative RT-PCR on two pairs of nasopharyngeal and oropharyngeal samples taken 24 hours apart.

Data was collected from patient treatment chart, patients were interviewed for missing data. EPI Info version 25 was used for data analysis. Ethical clearance was obtained from St Paul's Millennium Medical College Institutional Review Board(IRB).

## Results

This is a report of the first 33 COVID-19 patients in Ethiopia. The median age of the cases included in this study was 36 years (11 months to 84 years) with IQR of 22 years. The average length of hospitalization was 14 days (9 to 21 days). As shown in Table 1, the majority of the patients, 24/33(73%), were males, and 85% (n=28) were Ethiopians. Twenty-four patients (73%) had travel history to a country with confirmed COVID-19 cases, 6(18%) had contact with COVID-19 confirmed patients in Ethiopia with no travel history abroad. In three cases, there was neither travel history nor contact with COVID confirmed cases. Seventy-five percent of the patients (n=25) had mild illness at the time of admission. Five patients were admitted to intensive care units and four of them were mechanically ventilated. Three patients on mechanical ventilator died in the ICU, possible cause of death being sever hypoxia secondary to ARDS. Medical comorbidities were identified in 15% (n=5) of the cases included in this study. One case of COPD and another case of essential hypertension had mild COVID-19, both recovered. Two of the three deaths while diabetes and one had hypertension. Duration of intensive care unit stay ranged from 3 to 8 days. Those patients who died in the ICU stayed for an average of five days in the hospital. The oldest patient among the 33 cases in this report was 84 years old, had critical COVID with no comorbidity and was admitted to the ICU; discharged after full recovery.

**Table 1:** Socio-demographic characteristics of the first 33 COVID-19 patients admitted at Ekka-Kotebe Hospital, Addis Ababa, Ethiopia

Characteristics		Number	%
Age, years	<20	2	6.1
	21 to 30	8	42.2
	31 to 40	9	27.3
	41 to 50	8	24.2
	51 to 60	1	3.0
	61 and above	5	15.2
Gender	Male	24	72.7
	Female	9	27.3
Nationalities	Ethiopian	28	84.8
	Japanese	3	9
	Australia	1	3
	Mauritius	1	3
Occupation	Business man	14	48.3
	House wife	3	10.3
	Cabin crew	1	3.4
	Diplomat	1	3.4
	Pharmacist	2	7/0
	Nurse	2	7.0
	Security guard	1	3.4
	Student	1	3.4
	lawyer	1	3.4
	Unemployed	1	3.4
Travel History to	Driver	2	6.9
	No travel history	9	27.3
	Dubai	12	36.4
	Belgium	1	3.0
	Burkina Faso	1	3.0
	Congo Brazzaville	2	6.0
	London	1	3.0
	France	1	3.0
	Japan	1	3.0
	Canada	1	3.0
	Kenya	1	3.0
	Swaziland	1	3.0
	Thailand	1	3.0
	USA	1	3.0
Comorbidity	No comorbidity	28	84.8
	HTN	2	6.1
	DM	2	6.1
	COPD	1	3.0
Severity	Mild	25	75.8
	Critical	5	15.1%
	Moderate	3	9.1%
	Severe	0	0.0%

As shown on Table 2, the presenting symptoms in 70% of the patients was cough while nearly half of the patients had fever before or at the time of presentation to the hospital, and another half of the patients had headache. Diarrhea in 6(18%), sore throats in 5(15%) and dysuria in 2(6%) were among the rare symptoms in this study. Loss of taste and/or smell sensation was reported in 4(12%) patients. On laboratory investigations, 18(54.5%) patients had lymphopenia, 10/23(43.4%) of them had raised creatinine, and more than two third of the patients had raised AST and ALP.

**Table 2:** Major presenting symptoms of the first 33 COVID-19 patients admitted at Ekka- Kotebe Hospital, Addis Ababa, Ethiopia

Characterstics	Number	Percent
Cough	23	70
Headache	17	51.5
Fever	16	48.5
Myalgia	15	45.5
Diarrhea	6	18.2
Sour throat	5	15.2
SOB	5	15
Loss of taste and/or smell sensation	4	12
Fatigue	3	9
Dysuria	2	6

Chest X-ray was performed for ten patients. Six X-rays were reported normal, 2 patients had bilateral pleural effusion and additional 2 patients had bilateral diffuse infiltration. The 84 years old patient had chest CT with evidence of ARDS.

## Discussion

This study is the first COVID-19 report from Ethiopia. The study describes the demographic, clinical characteristics and treatment outcomes of the first 33 cases admitted to Ekka-Kotebe COVID-19 Treatment Center. Similar to reports from other countries outside China, this study showed that most of the cases in this study were either recent arrivals from abroad with the disease, 73%(n=24), or had contact with a person diagnosed to have COVID 19 (9). Similar to reports from Europe and China, males are more likely to develop the infection (7). The clinical profile of patients in this report range from mild symptom of upper respiratory infection to symptoms of organ damage. The clinical manifestation and disease severity at the time of admission in the literatures vary from country to country. Where cases are identified by community screening, most of the cases are either pre symptomatic or mild form. In countries where a large number of people are infected in a short period of time due to delay in introduction of public health preventive measures, screening and diagnosis of COVID is made mostly on symptomatic cases visiting healthcare facilities. In these areas, patients mostly present late after the initial symptom often times at stage of moderate to severe disease (11). The reason for more mild cases in this report may be due to the fact that most of our cases are diagnosed by screening from quarantine where people coming from abroad or those with contact history are isolated for two weeks. Like reports from Asia and Europe, the most frequent clinical symptom in our cases was cough, followed by headache and fever. Rare COVID-19 clinical symptoms reported in literatures, like loss of taste and/or smell sensations, were reported in four cases in this study (8–11). This indicates the need to look for very rare symptoms and symptoms not yet reported in COVID-19 confirmed cases, in order to increase the accuracy of clinical diagnostic criteria in settings where testing facility is limited.

Even though, in this study, nearly half of the cases who had organ function tests had abnormal renal and liver function test result, in all except three it resolved completely. This shows that laboratory evidence of organ damage alone may not be strong predictor of mortality.

All cases received Chloroquine sulphate, Azithromycin oral treatment and paracetamol for pain and fever. Oxygen therapy by face mask when oxygen saturation drops below 90% and by mechanical ventilator when patients are unable to maintain saturation with low flow oxygen treatment were vailable for the cases in the center. No antiviral medication, convalescent plasma or any traditional medicine was administered for any of the cases included in this report. Three patients died in the ICU while on mechanical ventilator, making the case fatality rate of 9%.

The first death was to a 60 years old known diabetic female patient on oral hypoglycemic agent. The patient had recent travel to France and she was diagnosed with COVID 19 five days after arrival to Ethiopia. She was transferred to ICU on second day of admission and was on mechanical ventilator for 5 days before death. Possible cause of death was severe hypoxia secondary to ARDS.

The second death was to a 65 years old hypertensive male patient who did not have travel or known contact history. He was diagnosed with COVID-19 while in non COVID hospital for respiratory problem. He was admitted to Ekka-Kotebe treatment center and transferred to ICU on second day of admission and was on mechanical ventilator for two days before death. Possible cause of death being severe hypoxia.

The third death was to a 65 years old, known diabetic female, who did not have known contact or travel history. She was diagnosed with COVID 19 while being treated in ICU in a non COVID hospital. She was transferred and admitted to Ekkakotebe COVID treatment center ICU with the diagnosis of critical COVID-19. She was on mechanical ventilator for three days before death. Possible cause of death is multiple organ damage, ARDS.

Most of the patients in this report were younger than 40 years with male predominance, and few had comorbidities. The deaths were 60 years and above. Except three, all cases in this report were either imported or had contacts with confirmed cases. The clinical distribution (mild, moderate, severe and critical) was as it is observed in most other countries. Cough was the commonest symptom followed by headache and fever. As it was in the early stage of the pandemic in Ethiopia, further observation of the new cases in the future will reveal the correct socio-demographic and clinical profile of COVID-19 in Ethiopia.

Ekka-Kotebe COVID-19 Treatment Center has limitation in providing portable chest X-ray for the critically ill patients. More lung pathologies could have been identified if CT scan was accessible in the center.

## Figures and Tables

**Table 3:** Laboratory results of the first 33 COVID-19 patients admitted at Ekka-Kotebe Hospital, Addis Ababa, Ethiopia

Characteristics		Number	Percent
Leucocyte count	High	1	3.0
	Low	12	36.4
	Normal	20	60.6
Lymphocyte count	High	3	9.1
	Low	18	54.5
	Normal	12	36.4
Platelet count	Low	3	9.4
	Normal	29	90.6
Creatinine	High	10	43.5
	Normal	13	56.5
AST	High	7	46.7
	Normal	8	53.3
ALT	High	3	20
	Normal	12	80
ALP	High	9	64.3
	Normal	5	35.7
